# Incidentally detected cavernous hemangioma of the glans penis after circumcision: A case report

**DOI:** 10.1097/MD.0000000000020217

**Published:** 2020-05-22

**Authors:** Kwang Seog Kim, Hyeok Lee, Jae Ha Hwang, Sam Yong Lee

**Affiliations:** Department of Plastic and Reconstructive Surgery, Chonnam National University Medical School, Gwangju, Korea.

**Keywords:** circumcision, glans penis, hemangioma

## Abstract

**Rationale:**

Hemangiomas of the glans penis are very rare. Treatment options include surgical excision, laser therapy, intralesional sclerotherapy, electrofulguration, and cryotherapy. However, there have been no definitive treatment guidelines established to date.

**Patient concerns:**

A 19-year-old man presented with a mass on the glans penis, incidentally found during a circumcision performed at a local urology clinic 3 months before visiting our department.

**Diagnoses:**

Histopathological examination identified the specimen as a cavernous hemangioma.

**Interventions:**

The mass was completely excised and the resulting wound was closed layer by layer.

**Outcomes:**

The patient was discharged without complications, such as wound dehiscence or infection. Follow-up 14 months after surgery showed that the wound was well healed without recurrence and the patient was satisfied with the aesthetic result.

**Lessons:**

Although there are many options to treat hemangiomas occurring on the glans penis, surgical excision can be considered when they are small in size.

## 1 Introduction

Hemangiomas may be of several types, including capillary, cavernous, compound, or lobular. They are the most common type of tumors in children, but only 2% of hemangiomas occur in the urinary tract. Even among urinary tract hemangiomas, cases where the tumor occurs on the glans penis are extremely rare.^[1]^ Asymptomatic cases are treated conservatively, while cases that cause functional and aesthetic issues can be treated using surgical excision, laser therapy, intralesional sclerotherapy, electrofulguration, or cryotherapy.^[2]^ However, no definitive treatment guidelines for hemangiomas of the glans penis have been established to date. In this article, we report a case involving the surgical excision of a hemangioma of the glans penis that was incidentally detected after circumcision.

## 2 Case presentation

**Figure 1:**
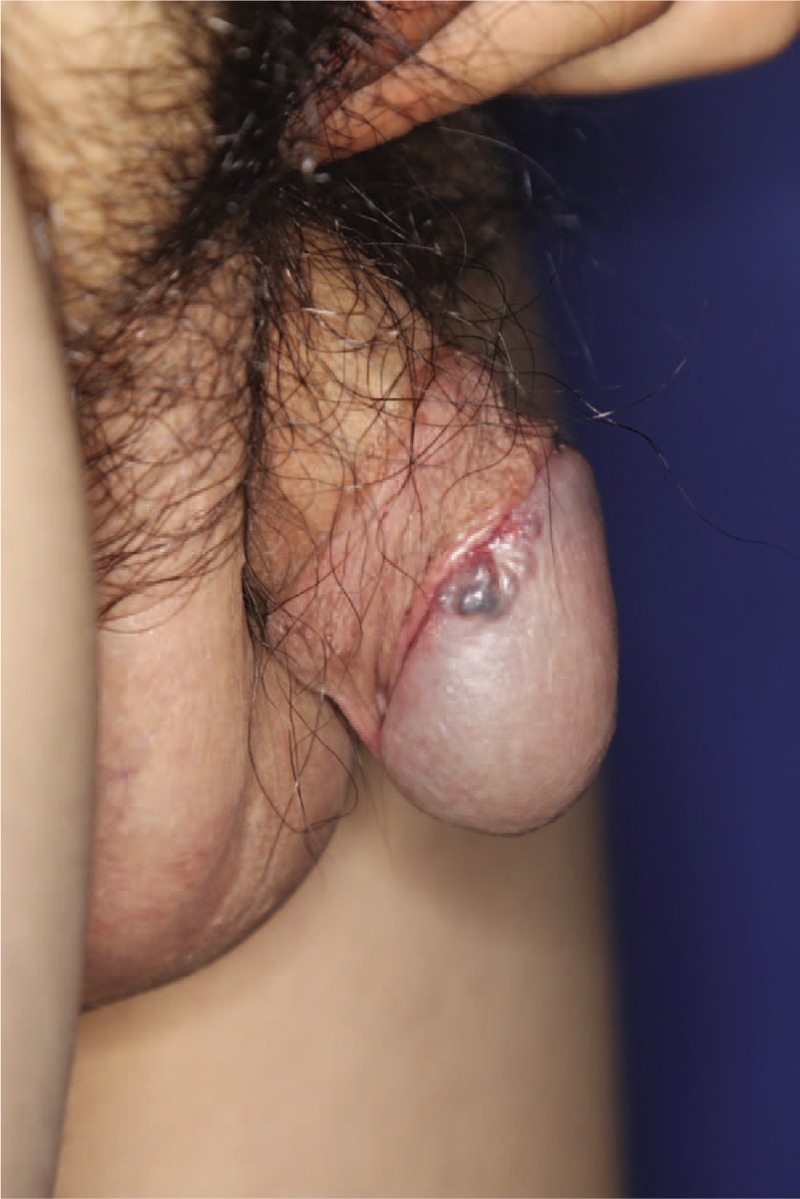
Preoperative photograph.

A 19-year-old man underwent circumcision at a local urology clinic 3 months before visiting our department. A mass was incidentally detected on the glans penis during the surgery, which was suspected to be a cyst. The patient underwent punch biopsy at a local dermatology clinic and the results were consistent with hemangioma. The patient and his parents desired surgical treatment rather than multiple procedures and presented to the outpatient clinic of our department. The mass at presentation was 1.0 × 0.8 cm in size, painless, non-tender, blue-red colored, and tortuous (Fig. 1). Since biopsy performed at the local dermatology clinic suggested a hemangioma, no imaging studies (such as ultrasonography or computed tomography) were performed.

**Figure 2:**
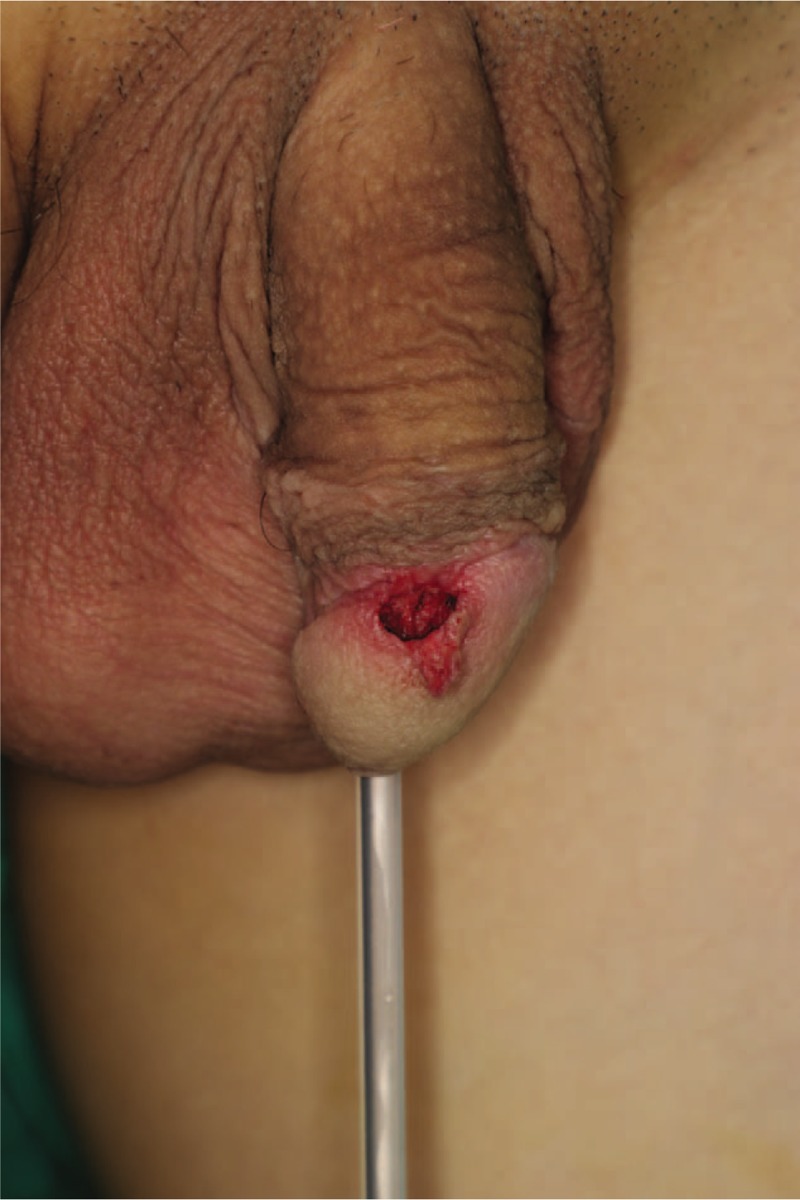
Intraoperative photograph.

**Figure 3:**
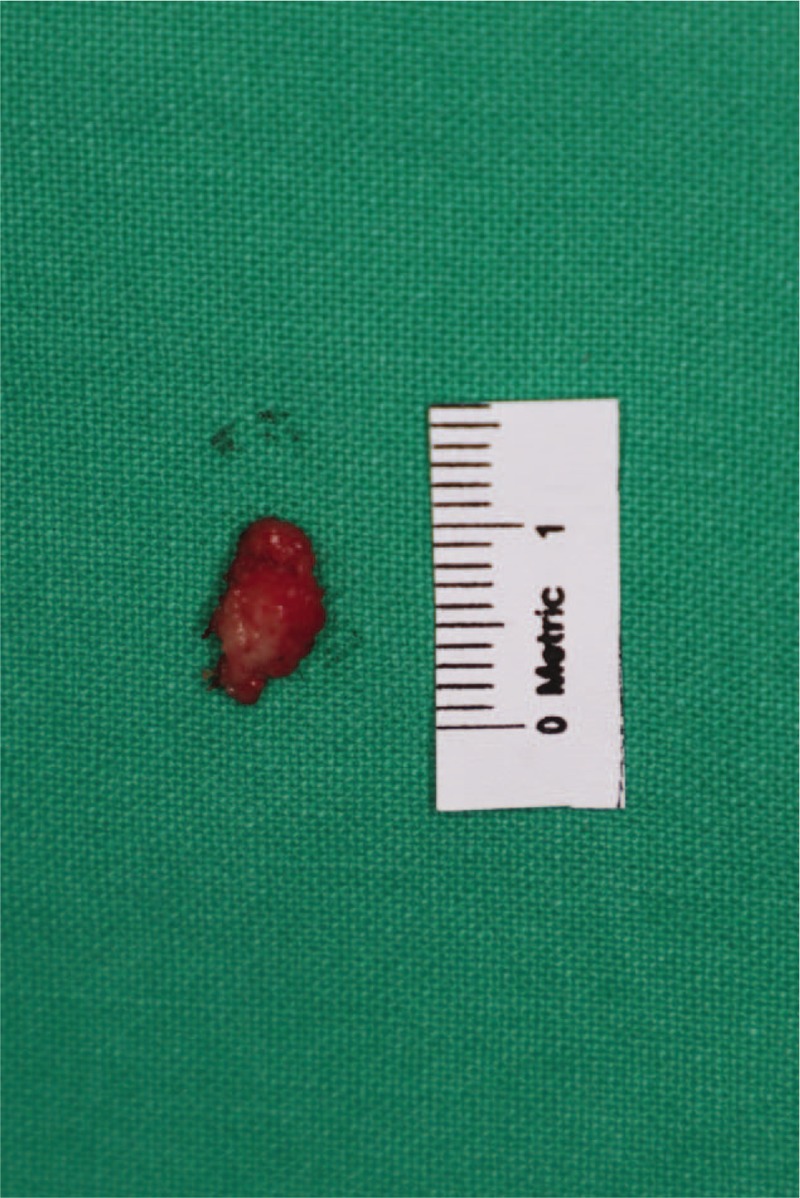
Surgical specimen.

**Figure 4:**
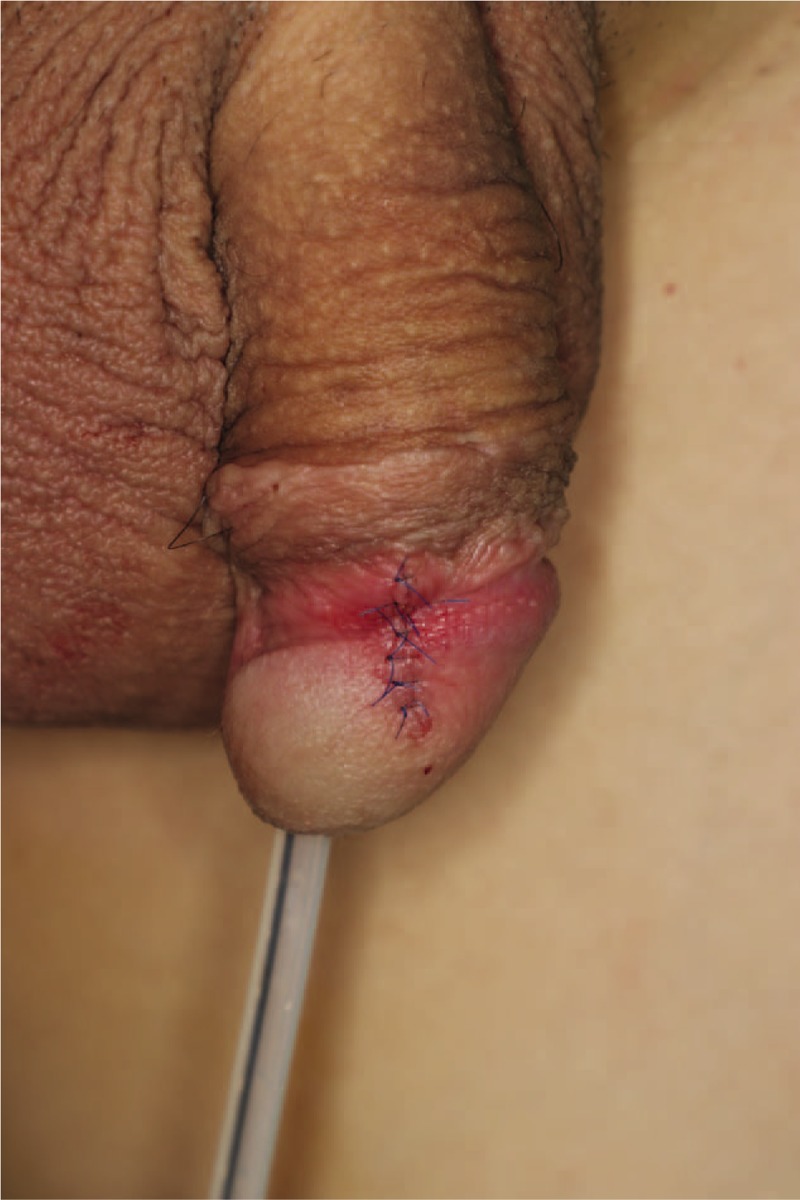
Immediate postoperative photograph.

The patient was placed in the supine position under general anesthesia. After the operative field was aseptically draped and the patient underwent Foley catheterization to prevent contamination, an elliptical incision line was drawn around the margin using a surgical marking pen. Local anesthesia was induced using 2% lidocaine mixed with epinephrine at a ratio of 1:100,000, which infiltrated the tissue around the mass. The mass was completely excised using a scalpel and bleeding was controlled using bipolar electrocautery (Figs. 2 and 3). Frozen biopsy was performed to completely rule out malignancy and showed a benign lesion. After confirming that there was no distortion of structures with the approximation of the skin flap, the resulting wound was closed layer by layer (Fig. 4).

**Figure 5:**
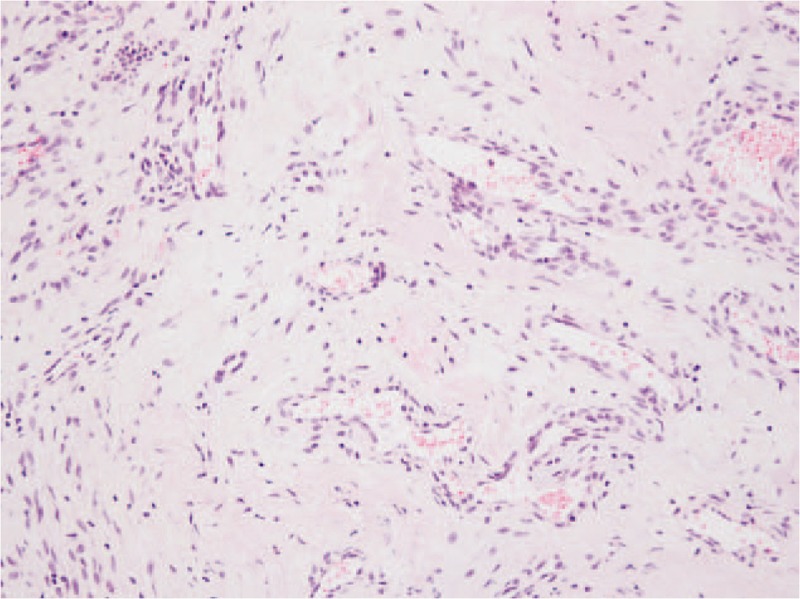
Photomicrograph shows classic features of a cavernous hemangioma, including dilated blood vessels lined with endothelial cells encapsulated by fibrous tissue and containing red blood cells (hematoxylin-eosin stain, × 200).

**Figure 6:**
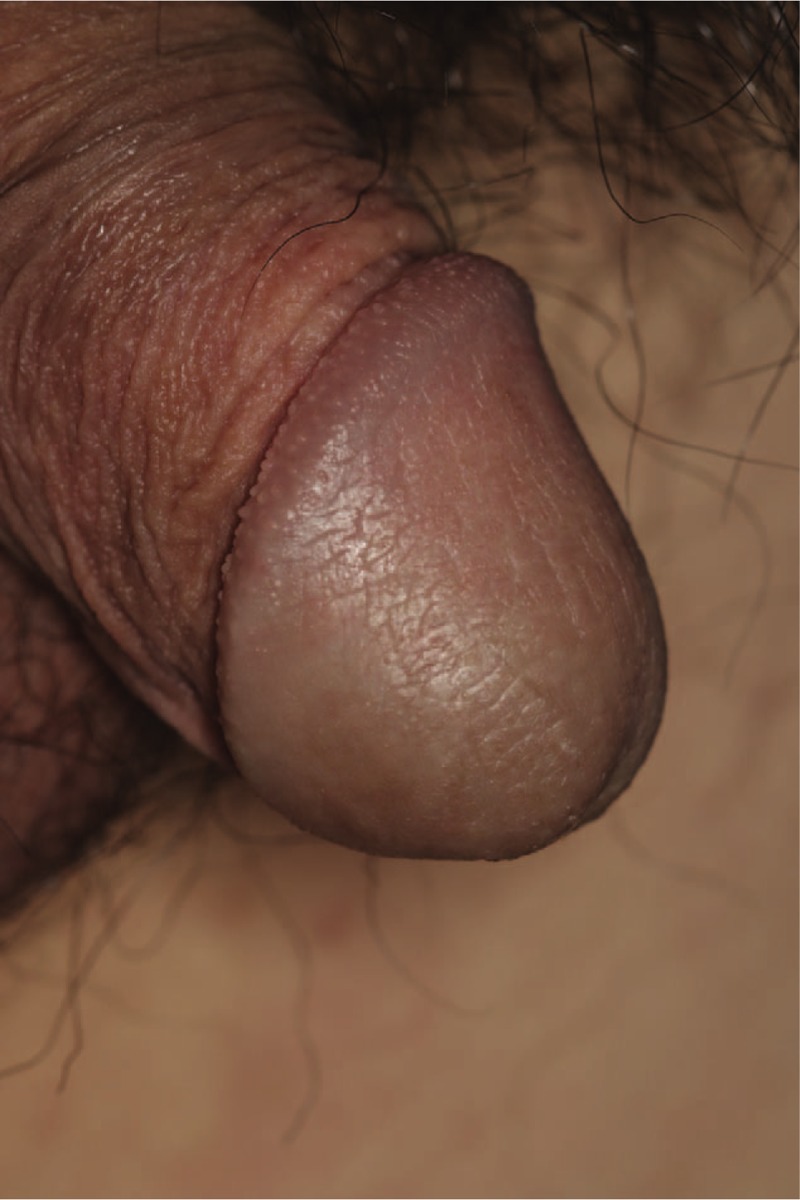
Photograph 14 months after surgery.

Permanent biopsy showed classic features of cavernous hemangioma, including dilated blood vessels lined with endothelial cells encapsulated by fibrous tissue and containing red blood cells (Fig. 5). Total stitch-out was performed 14 days after surgery, and the patient was discharged without complications, such as wound dehiscence or infection. Follow-up 14 months after surgery showed that the wound was well healed without recurrence and the patient was satisfied with the aesthetic result (Fig. 6).

The patient provided informed consent for the publication of his clinical and radiological data. This study was approved by the Institutional Review Board of Chonnam National University Hospital (CNUH-2018-227) and was conducted in accordance with the principles of the Helsinki Declaration II.

## 3 Discussion

Hemangiomas are the most common benign vascular tumors, but hemangiomas in the urogenital area are rare.^[3,4]^ Urogenital hemangiomas occur mostly in the kidney and bladder, and hemangiomas in the urethra, genital skin, and prostate have also been reported.^[3,5]^ However, hemangiomas occurring on the glans penis are especially rare.^[1]^ A hemangioma that remained hidden under the foreskin throughout childhood and was detected only after circumcision has not yet been reported. There is no standardized recommendation or treatment guideline for such hemangiomas owing to such a low incidence rate.^[6,7]^

Neodymium:yttrium–aluminum–garnet (Nd:YAG) lasers, first introduced by Jimenez-Cruz and Osca in 1993 for the treatment of hemangioma of the glans penis, emit a 1060-nm wavelength beam and promote the coagulation of the hemangioma tissue. Its advantages include excellent functional and aesthetic outcomes with minimal scarring.^[3,8]^ However, it is limited by the fact that it must be performed serially over a few months and the equipment required is expensive, which reduces patient accessibility and increases cost.^[5]^

Intralesional sclerotherapy is a widely used treatment approach in similar cases in developing countries owing to its cost-effectiveness.^[9,10]^ A sclerosant destroys the endothelium and triggers edema, resulting in thrombus formation in the vessel lumen and fibrosis. The lesion is subsequently removed by histologic absorption.^[11]^ However, despite its cost-effectiveness and the fact that it is easy to perform in outpatients, its use is steadily declining owing to associated complications such as cutaneous necrosis, ulceration, and hyperpigmentation.^[1]^

Surgical excision has traditionally been used for large, multiple hemangiomas that are difficult to treat non-surgically.^[12]^ In addition, unlike laser therapy or sclerotherapy, this option has the advantage of treatment completion in one visit. Thus, surgical excision can be performed for small, single lesions according to patient preference. However, it has a higher risk of bleeding and scar formation compared to non-surgical treatment.^[2,3]^

Because a glans penis hemangioma is very rare, there are no large-scale clinical studies of it and its prognosis is not well-known. There may be several factors that influence prognosis, including the type, growth, size, and location. They can continue to grow, but most of them do not and their sizes are relatively small compared to those of other sites.^[2,3]^ If their size is small, the prognosis after treatment is good regardless of the type or location.^[6]^ However, if they are large, they can only be removed by surgery, in which case aesthetic and functional impairments cannot be avoided.^[5]^

In the present case, it was difficult for the patient to receive multiple treatments because of his work schedule, and the patient and his parents desired rapid completion of treatment. Thus, surgical excision was performed, and good functional and aesthetic outcome was achieved without any complications.

In summary, when a hemangioma of the glans penis is small in size, surgical excision can be a good treatment option because it is a single-stage procedure and can provide an excellent aesthetic and functional outcome.

## Author contributions

**Conceptualization:** Kwang Seog Kim.

**Data curation:** Hyeok Lee.

**Formal analysis:** Jae Ha Hwang, Sam Yong Lee.

**Investigation:** Hyeok Lee.

**Methodology:** Jae Ha Hwang, Sam Yong Lee.

**Project administration:** Kwang Seog Kim.

**Writing – original draft:** Kwang Seog Kim, Hyeok Lee.

**Writing – review & editing:** Kwang Seog Kim.

Kwang Seog Kim orcid: 0000-0002-6766-4640.

Jae Ha Hwang orcid: 0000-0001-6992-8067.

Sam Yong Lee orcid: 0000-0002-3185-2519.
